# Antibodies with Higher Bactericidal Activity Induced by a *Neisseria gonorrhoeae* Rmp Deletion Mutant Strain

**DOI:** 10.1371/journal.pone.0090525

**Published:** 2014-03-04

**Authors:** Guocai Li, Rushan Xie, Xiaoping Zhu, Yanli Mao, Shuangxi Liu, Hongmei Jiao, Hua Yan, Kun Xiong, Mingchun Ji

**Affiliations:** 1 Department of Pathogen Biology and Immunology, Yangzhou University School of Medicine, Yangzhou, Jiangsu, China; 2 Jiangsu Key Laboratory of Zoonosis, Yangzhou University, Yangzhou, Jiangsu, China; 3 Department of Pathogen Biology, Heze Medical College, Heze, Shandong, China; 4 Clinical Testing Center, College of Clinical Medicine, Yangzhou University, Yangzhou, Jiangsu, China; 5 Department of Anatomyand Neurobiology, Central South University School of Basic Medical Sciences, Changsha, Hunan, China; CNR, Italy

## Abstract

*Neisseria gonorrhoeae* (*N. gonorrhoeae*) outer membrane protein reduction modifiable protein (Rmp) has strong immunogenicity. However, anti-Rmp antibodies block rather than preserve the antibacterial effects of protective antibodies, which hampers the development of vaccines for gonococcal infections. We herein constructed an Rmp deletion mutant strain of *N. gonorrhoeae* by gene homologous recombination. The 261–460 nucleotide residues of Rmp gene amplified from *N. gonorrhoeae* WHO-A strain were replaced with a kanamycin-resistant Kan gene amplified from pET-28a. The resultant hybridized DNA was transformed into *N. gonorrhoeae* WHO-A strain. PCR was used to screen the colonies in which wild-type Rmp gene was replaced with a mutant gene fragment. Western blotting revealed that the Rmp deletion mutant strain did not express Rmp protein. Rmp deletion did not alter the morphological and Gram staining properties of the mutant strain that grew slightly more slowly than the wild-type one. Rmp gene mutated stably throughout 25 generations of passage. Antibody-mediated complement-dependent cytotoxicity assay indicated that the antibodies induced by the mutant strain had evidently higher bactericidal activities than those induced by the wild-type strain. Further modification of the Rmp deletion mutant strain is still required in the development of novel live attenuated vaccines for gonorrhea by Opa genes deletion or screening of phenotypic variant strains that do not express Opa proteins.

## Introduction

*Neisseria gonorrhoeae* (*N. gonorrhoeae*) infects approximately 106 million people worldwide annually as estimated by the World Health Organization [1]. Upon infection, *N. gonorrhoeae* may spread to the fallopian tubes, pelvic cavity and other parts of female patients, leading to serious consequences such as infertility or ectopic pregnancy [2]–[3]. Besides increasing the risks of HIV transmission [4]–[5], *N. gonorrhoeae* burdens the treatment of gonorrhea owing to the high frequency of acquired resistance to multiple antibiotics [6]. Therefore, vaccines are an attractive option for preventing gonorrhea [7].

A variety of surface antigens, e.g., pilus [8]–[12], lipooligosaccharide (LOS) [13]–[15], opacity-associated protein (Opa) [16], porins [17]–[19], transferrin-binding proteins [20]–[21], surface protein A (NspA) [22]–[23], lipoproteins [24], outer membrane preparations [25], have been used to develop vaccines for *N. gonorrhoe*ae. Two of these antigens have been in clinical trials and few have been tested in animal models. Based on the current studies regarding *N. gonorrhoeae* vaccines, it may be difficult to completely prevent *N. gonorrhoeae* infections by vaccines comprising only one type of antigen, which may be associated with the complicated compositions of *N. gonorrhoeae* antigens and the unsuccessful use of individual antigens to elicit gonococcal immunity hitherto [7]. Therefore, we speculate that vaccines containing more or even all the protective antigens of *N. gonorrhoeae* may be optimum.

Among *N. gonorrhoeae* antigens, outer membrane protein reduction modifiable protein (Rmp) is identified to play a role of immunosuppression. Rmp was discovered in the study of porin subunit vaccines, is ubiquitously expressed in *N. gonorrhoeae* almost without variation as a highly conserved protein [26]–[28]. The *rmp* gene deduced amino-acid sequence shows a coding frame of 236 amino acids consisting of the known NH_2_-terminal sequence of Rmp and a typical 22-amino-acid signal peptide [29]–[30]. Being about 30–31 kDa after SDS-PAGE electrophoresis [31]–[32], Rmp is a good antigen with immunogenicity higher than porin, and is able to stimulate the production of complement-binding antibodies. However, killing of *Neisseria gonorrhoeae* by immune serum is prevented or blocked by purified IgG antibodies against Rmp. Immune convalescent serum from the patients recovering from disseminated gonococcal infection without bactericidal activity is restored by selectively depleting Rmp antibodies using immunoabsorption, indicating that Rmp antibodies in normal and immune human sera play an important role in serum resistance of *N. gonorrhoeae* [32]. Rmp antibodies even can increase the susceptibility to *N. gonorrhoeae* infection. By studying the relationship between Rmp antibody and *N. gonorrhoeae* mucosal infection, Plummer et al. [33] found that the Rmp antibody levels in Nairobi prostitutes were positively correlated with the risk of *N. gonorrhoeae* infection. Women with positive Rmp antibody are more prone to infection than those are negative (OR = 3.4, P<0.05), suggesting that Rmp antibody is capable of increasing the susceptibility to *N. gonorrhoeae* mucosal infection. To circumvent the reduction of vaccine protective efficacy owing to Rmp contamination in purifying Por vaccine, Wetzler et al. [34] constructed *Neisseria gonorrhoeae* lacking Rmp in its outer membrane using gonococcal strain F62. The mutant strain 2D can be used to study the role of Rmp in gonococcal physiology, metabolism, membrane structure, and pathogenesis, thus allowing purification of gonococcal proteins without Rmp contamination.

We herein propose that Rmp deletion mutant strain which does not express Rmp protein could induce antibodies with higher bactericidal activity since wild-type *N. gonorrhoeae* possesses an immunosuppressing gene *rmp* encoding Rmp that generates undesirable blocking antibodies. Therefore, the mutant strain is a promising candidate for novel attenuated live vaccines for gonorrhea. In this study, an Rmp deletion mutant strain using WHO-A as background strain was constructed by homologous recombination, the biological stability and growth characteristics of which were studied. Besides, the antibodies induced by the mutant and wild-type strains were compared in reference to their antibacterial activities against *N. gonorrhoeae*. The results show that the mutants are potential tools in developing new attenuated live vaccines for the prevention of *N. gonorrhoeae* infections.

## Methods

### Ethics Statement

All experimental protocols on mice were carried out according to the principles outlined in the NIH Guide for Care and Use of Laboratory Animals (NIH Publication No. 85–23, Revised 1996). This study was approved by Animal Ethics Committee of Yangzhou University.

### Construction of *rmp* mutant gene

**Figure 1:**
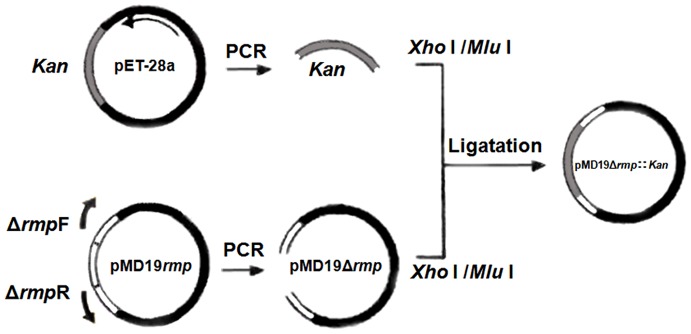
Construction procedure of pMD19▵*rmp*::*Kan*. After subcloning Rmp gene in pMD19-T, forward primer was designed in the 3′-flanking region, and reverse primer was designed in the 5′-flanking region. PCR was conducted to obtain DNA fragment pMD19▵*rmp* with flanking regions in both terminals, which was thereafter connected with Kan gene treated with the same restriction endonucleases, yielding inactivated gene ▵*rmp*::*Kan* with the intermediate portion of Rmp gene replaced with Kan gene.

*N. gonorrhoeae* strain WHO-A was described previously [23], from the genomic DNA of which pMD19-rmp was obtained by amplifying gene of *rmp* and connecting it with pMD19-T utilizing *rmp*F and *rmp*R as the forward and reverse primers respectively. Then forward primer Δ*rmp*F was designed in the 3′-flanking region of *rmp*, and reverse primer Δ*rmp*R was designed in the 5′-flanking region. PCR was carried out by using recombinant vector pMD19-*rmp* as the template to acquire pMD19▵*rmp* with the middle 261–460 nucleotide residues of *rmp* truncated that was then connected with kanamycin-resistant gene *Kan* amplified by PCR using *Kan*F and *Kan*R as the forward and reverse primers from pET-28a, yielding recombinant vector pMD19▵*rmp*::*Kan*. The primers used in construction are summarized in Table 1, and the detailed procedure is shown in Fig. 1.

**Table 1:** Primers used to construct pMD19▵*rmp*::*Kan.*

Primer	Primer sequence (5′-3′)	Introduced restriction endonuclease
*rmp*F	CGGGATCCATGACCAAACAGCTGAAATTAAG	*Bam* H I
*rmp*R	CCCAAGCTTTTAGTGTTGGTGATGATTGCGT	*Hind* ▵
▵*rmp*F	TGCCTCGAGTAGTGGCAAACAACCTGGTC	*Xho* I
▵*rmp*R	GTCACGCGTTTATCTACTCAACATATTGAGGAGCCTGC	*Mlu* I
*Kan*F	TCGACGCGTGCTCAGTGGAACGAAAACTC	*Mlu* I
*Kan*R	GCGCTCGAGCTTAGAAAAACTCATCGAGCAT	*Xho* I

Thereafter mutant gene fragment ▵*rmp*::*Kan* was cut off from pMD19▵*rmp*::*Kan* by *Bam*H I and *Hind* III (Fermentas China Co., Ltd., Shenzhen, Guangdong, China), which was then purified and electrotransformed into *N. gonorrhoeae* WHO-A strain. *N. gonorrhoeae* transformants were screened on GC chocolate agar plates containing kanamycin, from which single colonies were selected, cultured, and PCR-amplified with *rmp* and *Kan* primers. The homozygous mutant strains of *N. gonorrhoeae* were screened with wild-type gene of *rmp* being substituted by the mutant one.

### *Western blotting*

Cloned full length *rmp* gene was inserted into pET-30a, and the resultant recombinant expression vector pET-30a-*rmp* was transformed into *E. coli* BL21 (DE3) strain. SDS-PAGE analysis was performed to detect the expression of recombinant Rmp (rRmp) following the induction of bacteria for 6 h by 1 mmol/L IPTG. Ni-NTA Superflow (Qiagen Inc., Valencia, California, USA) was used to purify rRmp according to the manual. After renaturing rRmp, mice were immunized with the protein to prepare anti-Rmp antibodies. Five 6-week-old female BALB/c mice were selected and subject to abdominal subcutaneous multi-point injection with renatured recombinant Rmp (rrRmp, 100 µg per mouse). Second immunization was performed two weeks after the first one, and booster immunization was performed 3 times once every two weeks. Serum was collected on the 5th day after the 4th immunization. The titer of anti-Rmp was detected by indirect ELISA using rrRmp and wild-type strain of *N. gonorrhoeae* as antigen, respectively.

The cultures of Rmp deletion mutant strain and wild-type strain with similar bacterial contents were collected separately, washed with PBS twice, and resuspended in 50 µl of PBS, into which 5×SDS sample buffer was added *pro rata*. The resulting suspensions were mixed evenly and boiled at 100°C for 10 min. The lysates were taken for SDS-PAGE, transferred to PVDF membrane to detect the deletion of Rmp expression in the mutant strain by Western blotting utilizing Rmp immune serum as the primary antibody.

### *Observation of morphological and growth characteristics*

The mutant and wild-type strains were subject to Gram staining, respectively, and their morphological characteristics were observed with oil-immersion lens.

The Rmp deletion mutants were subcultured and the 5th, 10th, 15th, 20th and 25th generation ones were eluted by FB liquid culture medium [35]–[36]. The OD_600_ values of the eluted bacterial suspensions were adjusted to 1.0, and then viable cells were counted on ordinary GC chocolate agar plates and those containing 40 µg/mL kanamycin, respectively.

The Rmp deletion mutant and WHO-A wild-type strains were cultured to the logarithmic growth phase, the OD_600_ values of which were adjusted identical in FB liquid culture medium. 5 ml of FB liquid culture medium and 5% of the two bacterial suspensions as inoculums were added into 15 ml test tubes, respectively. They then underwent stationary liquid culture in a 5% CO_2_ incubator at 37°C. The OD_600_ values of the bacterial cultures were measured by spectrophotometer hourly. The growth curves of the mutant and wild-type strains were plotted with measurement times as the X-coordinate and OD_600_ values as the Y-coordinate.

### *Antibody-mediated complement-dependent bacteriolysis experiment*

Cultures of the Rmp deletion mutant and wild-type strains were diluted with PBS buffer and the OD_600_ values of the bacterial suspensions were adjusted to 1.0. Two groups of 5 to 6-week-old female BALB/c mice (18–20 g) were selected and subject to abdominal subcutaneous multi-point injection of the two bacterial suspensions (100 µg per mouse). Second immunizations were performed two weeks after the first one, and booster immunizations were performed once every two weeks for four times in total. Mice blood was collected on the 5th day after the 4th immunization to prepare antiserum. Purified rrPorB was prepared by using the gonococcal PorB prokaryotic expression vector pUNCH682 gifted by Dr. Christophe [19]. Anti-PorB serum was prepared by the methods similar to that for preparing anti-Rmp serum.

The *N. gonorrhoeae*-specific antibacterial effects of the antisera of wild-type and mutant strains were compared by the previously described antibody-mediated complement-dependent bacteriolysis experiment [15], [18], [20], [23]. Briefly, *N. gonorrhoeae* stored at –80°C were recovered on GC chocolate agar plate. After being cultured in 5% CO_2_ incubator at 37°C for 16–18 h, several bacterial colonies were picked and inoculated in FB liquid media to be cultured for approximately 18 h, and the OD_600_ of the bacterial suspension was adjusted to 1.0. The immune sera of each mice group were collected and inactivated at 56°C for 30 min. 50 µl of serially diluted serum and 40 µl of bacterial suspension were mixed and incubated at 37°C with 5% CO_2_ for 15 min. After adding 10 µl of fresh human serum that originated from a healthy volunteer and did not react with gonococcal WHO-A strain as the complement. The suspension was incubated for another 45 min. The mixture was serially diluted and laid on GC chocolate agar plates. The number of colonies was counted after 18 h of culture. The *N. gonorrhoeae* incubated with normal mouse serum was used as the negative control group. Immune serum was defined as having significant bactericidal effects when the number of colonies of the experimental group was lower than 50% of that of the negative control group.

### *Statistical analyses*

Analysis of variance was performed using the software SPSS, version 10.0. One-way ANOVA analysis was performed to reveal means with standard deviation. LSD was used to address pairwise comparisons following the test of homogeneity of variances. A P value of less than 0.05 was considered significant.

## Results

### *Mutation of* *rmp* gene

Based on the published *N. gonorrhoeae rmp* gene sequences [30]–[31], forward (*rmp*F) and reverse (*rmp*R) primers for gene amplification were designed, and *rmp* gene of WHO-A strain was amplified by PCR with genomic DNA as the template (GenBank entry ID: HQ589134.1). Sequence analysis showed that *rmp* gene of WHO-A strain sequence was lengthed 711 bp, which shared approximately 99% homology with those of other gonococcal strains in GenBank.

**Figure 2:**
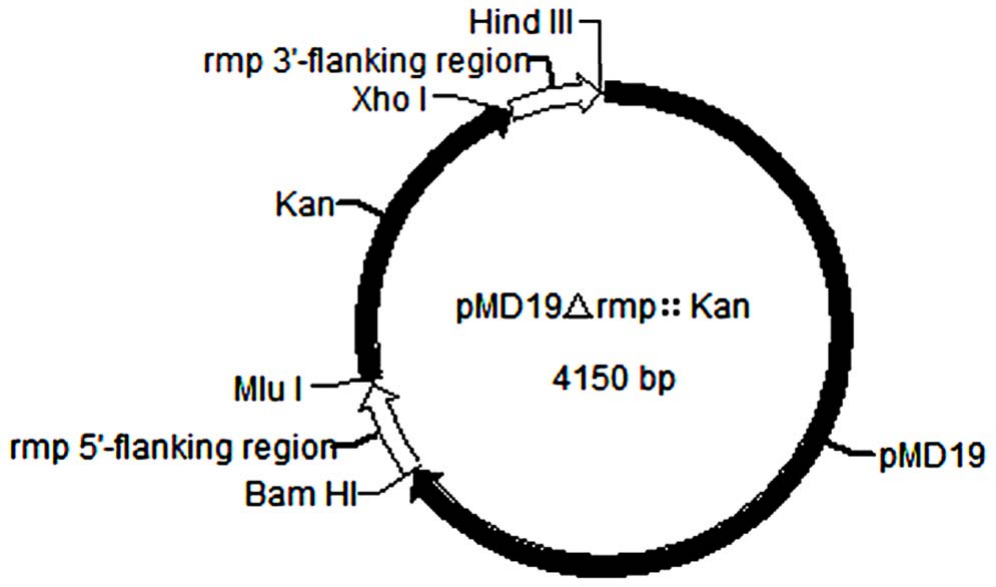
Schematic diagram of *rmp* mutant gene structure. Inactivated gene Δ*rmp*::*Kan* was acquired by substituting the 261–460 nucleotide residues of *rmp* gene for kanamycin-resistant gene, which was then subcloned in pMD19-T vector, yielding pMD19Δ*rmp*::*Kan* eventually.

After subcloning *rmp* in pMD19-T, using recombinant vector pMD19-*rmp* as the template, forward primer Δ*rmp*F in the 3′-flanking region and reverse primer Δ*rmp*R in the 5′-flanking region were utilized for PCR amplification to produce DNA fragment pMD19Δ*rmp* with 261–460 nucleotide residues missing in *rmp*. Kanamycin-resistant gene *Kan* was amplified with vector pET-28a as the template as well as *Kan*F and *Kan*R as primers. Connecting pMD19Δ*rmp* with *Kan* that had been treated with the same restriction endonucleases of *Xho* I and *Mlu* I generated recombinant plasmid pMD19Δ*rmp*::*Kan* (Fig. 2), i.e. *rmp*-inserted inactivated gene of Δ*rmp*::*Kan* with 261–460 nucleotide residues substituted by *Kan* gene.

### *Screening and identification of Rmp deletion mutant strain*

**Figure 3:**
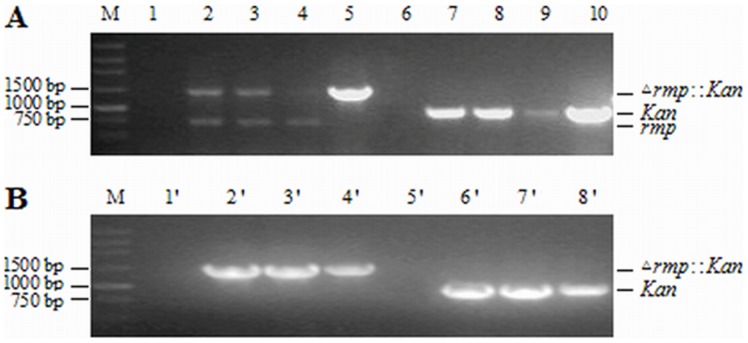
PCR identification of *N. gonorrhoeae* Rmp deletion mutants in the 1st (A) and the 7th (B) generation of transformants. The cultures of relevant strains were amplified using *rmp* primers (*rmp*F and *rmp*R) and Kan primers (*Kan*F and *Kan*R), respectively. Lanes 1, 6, 1’ and 5’ represent negative controls using pMD19-T as templates; lanes 5, 10, 4’ and 8’ represent positive controls using pMD19Δ*rmp*::*Kan* as templates.

pMD19Δ*rmp*::*Kan* was digested with *Bam*H I and *Hind* III to produce DNA segment Δ*rmp*::*Kan* which was electrotransformed in WHO-A strains. Then *Kan*-resistant *N. gonorrhoeae* strains were screened by Kan^+^ plate. Using the culture of 1st generation strains as the template, *rmp* primers were PCR-amplified and screened. Given that wild-type and mutant *rmp* genes were amplified simultaneously (Fig. 3A), wild-type *rmp* gene had not been entirely substituted. The amplification with *Kan* primers suggested existence of the gene in the transformant cells. PCR amplification was conducted with *rmp* primers by using the culture of 7th-generation transformants as the template. The results showed that only *rmp* mutant gene was amplified (Fig. 3B), verifying the production of *N. gonorrhoeae* Rmp deletion mutant strains.

**Figure 4:**
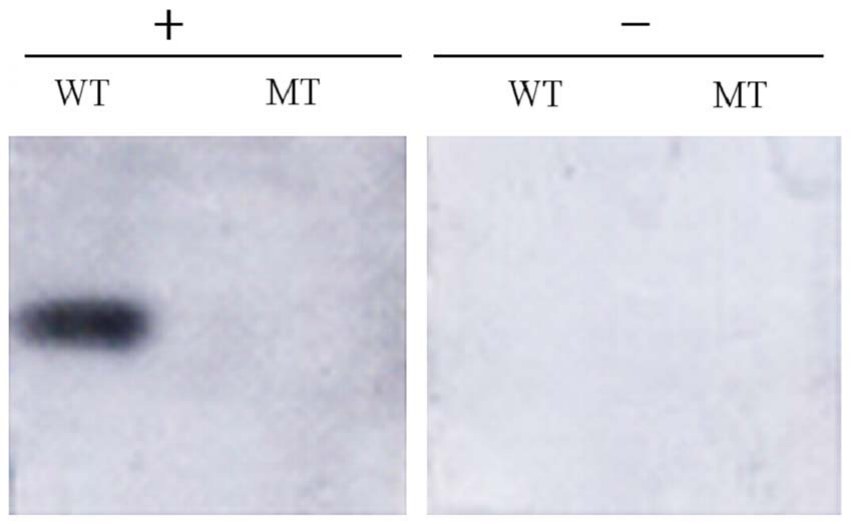
Western-blotting analysis of the expression of Rmp protein in wild-type and Rmp deletion mutant strains of *N. gonorrhoeae*. Lysates of wild-type (WT) and mutant (MT) strains were subjected to SDS-PAGE and were transferred to PVDF membranes to react with anti-rrRmp (+) and normal mouse serum (–), respectively.

Western-blotting detection was carried out with the lysates of *N. gonorrhoeae* wild-type and Rmp deletion mutant strains as the antigens and with mice rrRmp immune sera as the primary antibodies, respectively. We found that wild-type strain expressed approximately 30.0 kDa Rmp, while no protein band was detected in the corresponding location of the mutant strain, indicating that it failed to express Rmp. Notably, the serum of normal mice could not react with Rmp expressed by the wild-type strain (Fig. 4).

### *Morphological characteristics of Rmp deletion mutant strain*

The passaged Rmp deletion mutant and wild-type strains, which were subject to Gram staining and were observed with oil-immersion lens, were kidney-shaped Gram-negative diplococci without differed morphological or staining characteristics.

**Figure 5:**
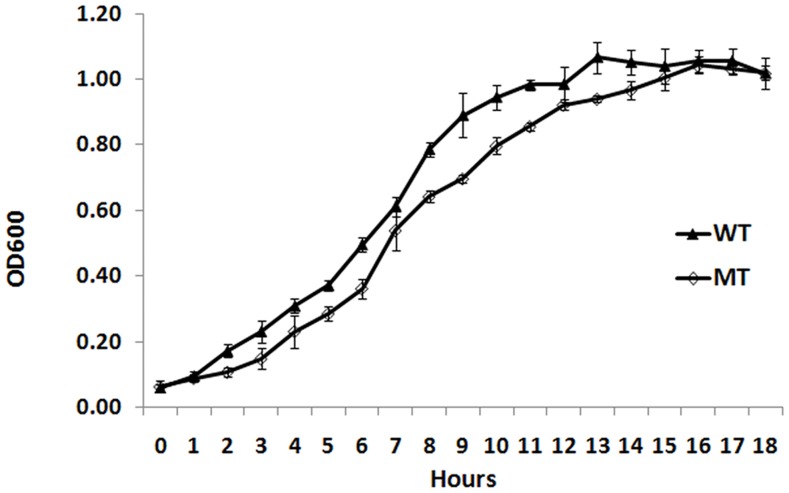
Growth curve of *N. gonorrhoeae* Rmp deletion mutant and wild-type strains. Same amounts of Rmp deletion mutant and wild-type strains were inoculated to FB liquid culture medium, from which 10 µl was sampled to measure the OD_600_ value every one hour.

Same amounts of Rmp deletion mutant and wild-type strains with identical OD_600_ values were inoculated to FB liquid culture medium, 10 µl of which was sampled to measure OD_600_ every one hour and to plot the growth curve. Fig. 5 exhibited that the Rmp deletion mutant strain grew slightly slower than the wild-type one.

### *Genetic stability of Rmp deletion mutant strain*

**Figure 6:**
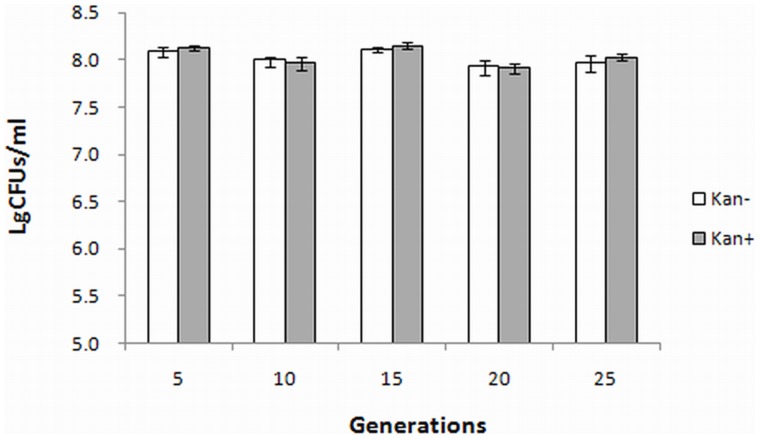
Detection of the genetic stability of *N. gonorrhoeae* Rmp deletion mutant strain. Same amounts of every 5 generations of culture products subcultured in ordinary culture medium were inoculated in FB liquid culture media with and without kanamycin for counting (n = 5).

Rmp deletion mutant strain was subcultured with antibiotic-free medium and the 5th, 10th, 15th, 20th and 25th generation products were eluted with FB liquid culture medium, respectively. The eluted bacterial suspensions were then subject to viable cells counting with ordinary or Kan^+^ GC chocolate agar plates. The results showed that the normally subcultured Rmp deletion mutant strain grew similarly on the two types of plates (Fig. 6), prompting that mutated *rmp* gene could be stably passaged concomitantly with host bacteria.

### *N. gonorrhoeae-*specific bactericidal activity of the antibodies against Rmp deletion mutant strain

**Figure 7:**
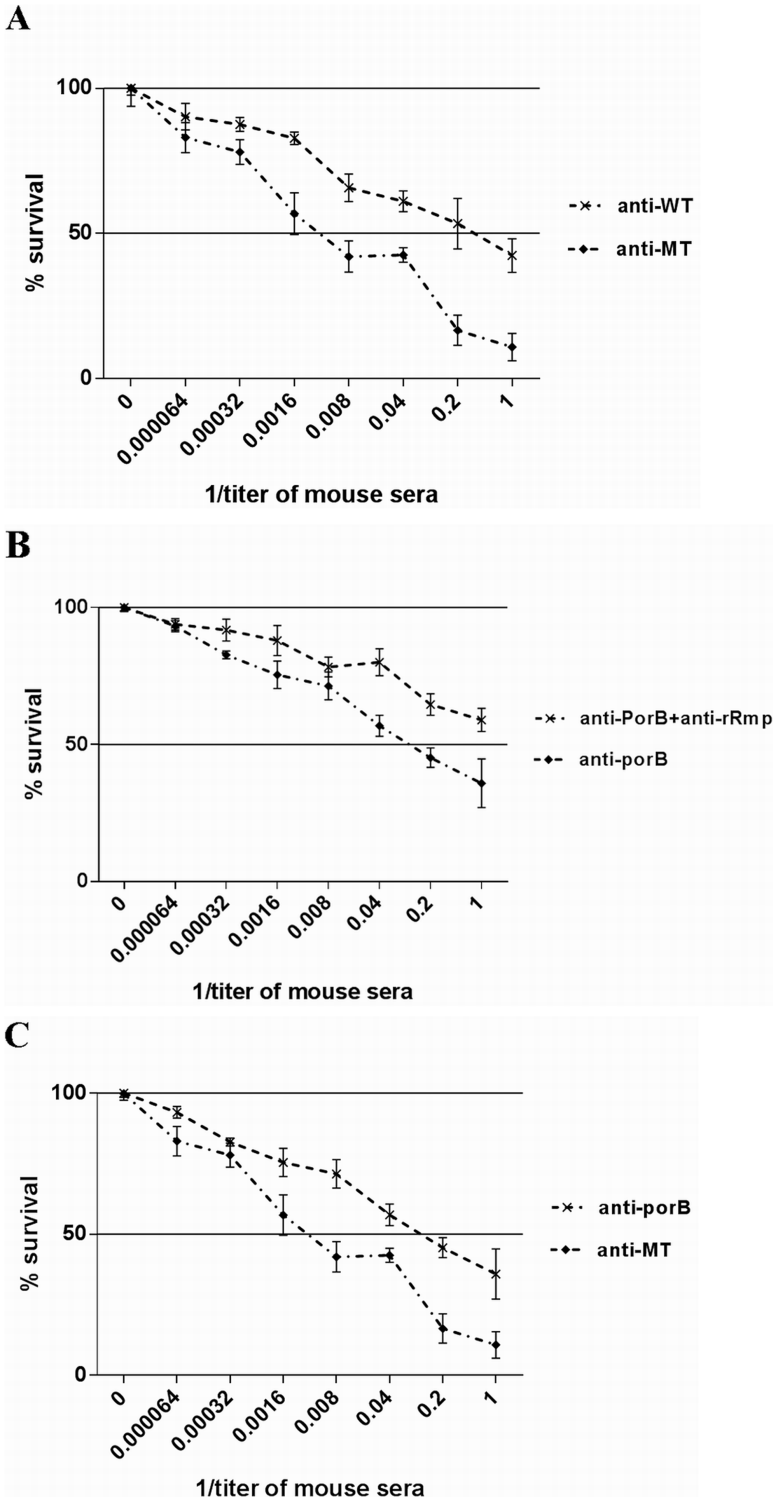
*N. gonorrhoeae-*specific bactericidal activity of relevant antibodies detected using antibody-mediated complement-dependent bactericidal activity assay. 50 µl of serially diluted antiserum and 40 µl of bacterial suspension were mixed and incubated for 15 min. After adding 10 µl of fresh human serum as the complement, the suspension was incubated for another 45 min. The survival cells in the mixture were counted on GC chocolate agar plates. Relative survival rate was derived from the ratio of the number of colonies in the antiserum incubation group to that in the normal serum incubation group, and antiserum was defined as having significant bactericidal effects in case the rate was lower than 50%. For all groups, n = 4. Error bars were drawn as mean with standard deviation.

Antibody-mediated complement-dependent bacteriolysis experiment, which can reflect the *N. gonorrhoeae*-specific antibacterial activity of the corresponding antibodies in the presence of complement, has been commonly used as the index for the *in vitro* evaluation on the protectiveness of gonococcal vaccines [15], [18], [20], [23]. The antisera of wild-type and mutant strains were mixed with wild-type *N. gonorrhoeae* WHO-A strain and incubated for 15 min, respectively, into which was added fresh human serum as the source of complement, followed by another 45 min of incubation. Then the number of survival colonies in the mixture was detected by plate counting. Significant bactericidal effect of inhibiting >50% gonococcal survival was discerned when the 1/titer of the Rmp deletion mutant strain antiserum was ≥0.008. The bacteria survival rate of the mutant strain antiserum group was significantly lower than that of the wild-type one at each dilution of sera 1/titer ≥0.008 (P < 0.05) (Fig. 7A), suggesting that the Rmp deletion mutant strain induced effective antibody immunity without Rmp immune repression [32]. To further reveal the immunity-blocking effect of Rmp antibodies, we used the mixture of rrRmp and rrPorB immune sera for complement-mediated bactericidal experiment. The mixed sera did not kill 50% of gonococci while rrPorB antiserum did when the serum 1/titer was ≥0.2 (Fig. 7B). The Rmp deletion mutant strain antiserum showed higher bactericidal effect than rrPorB antiserum at each sera dilution (1/titer ≥0.008) with significant bactericidal effect (P < 0.05) (Fig. 7C). The raw data and the P-values for 3 of the pairwise comparisons for Figure 7A, B and C are provided as supplementary material (Tables S1 and S2).

## Discussion

To develop a subunit vaccine of purified PI (porin) without the contamination of Rmp by using *N. gonorrhoeae* lacking Rmp in its outer membrane, Wetzler et al. [34] inserted beta-lactamase (beta la) in the *Xba* I site of Rmp gene, cut off Rmp/beta la insert by *Eco*R I and transformed it into *N. gonorrhoeae* F62 strain, yielding a Rmp deletion mutant strain 2D. To explore the feasibility of developing a novel attenuated live vaccine for gonorrhea by using immunosuppressive genes deletion gonococcal mutants, we replaced the 261–460 nucleotides of Rmp gene with Kan gene, and transformed Δ*rmp*::*Kan* into WHO-A strain. The mutant strains in which wild-type *rmp* genes were substituted by mutant ones were screened by PCR. The mutant strains did not express Rmp as suggested by Western blotting. In this study, the portion of *rmp* that has been excluded from the Rmp deletion mutant strain did not include the upstream portion of the gene (nucleotide residues 211–260) that encodes for the disulfide loop (aa 47–63) that is reported to be the major target site for blocking antibodies [37]. The negative Western blot indicated that the upstream portion was not expressed on this strain since the recombinant rRmp used to raise anti-Rmp antibody was itself full length Rmp.

Microscopic observation after Gram stain showed that Rmp deletion mutant and wild-type strains had identical morphological and staining characteristics. The subculture of gene deletion mutants may suffer from unstable homologous recombination, and loss of introduced resistance genes. In this study, *rmp* mutant gene remained intact throughout the 25 generations of passage as indicated by bacteria counting. In the meantime, the mutant strain grew slightly more slowly than the wild-type strain did, which was similar to the growth features of F62 Rmp deletion mutant strain 2D [34].

Moreover, we performed antibody-mediated complement-dependent bacteriolysis experiments to explore the potential application of Rmp deletion mutant strain in the development of gonorrhea vaccines. The antiserum of the mutant strain exerted obviously more potent bactericidal effects against *N. gonorrhoeae* than that of wild-type strain, suggesting that antibodies against Rmp induced by the wild-type strain in the antiserum might inhibit the bactericidal effects of protective antibodies [32]. Adding rrRmp antiserum did reduce the antibacterial activity of rrPorB antiserum, inferring that Rmp antibodies inhibited the bactericidal activity of effective antibodies, and that OM protein Rmp was one of the molecular mechanisms on which *N. gonorrhoeae* rely to escape the bactericidal effect of human immune system [32]–[33]. Antiserum against whole cell antigens of the mutant strain was more bactericidal than antibody against single antigen of rrPorB, indicating that attenuated live vaccines would be more promising in the control of gonorrhea.

Gotschlich et al. [30] published the DNA sequence of Rmp gene of *N. gonorrhoeae* R10 strain, and submitted it to GenBank in April 2005. We cloned the Rmp genes of WHO-A strain (HQ589134.1) and a clinical isolate YZ06 (HQ589132.1) with exactly the same ORF sequences, which shared 100% homology with the ORF sequence of Rmp gene (X05105.1) of R10 strain [30], Omp3 gene of TCDC-NG08107 (CP002440.1) [38] and NCCP11945 (CP001050.1) [39], shared 99% homology with the ORF sequence of Omp3 gene of FA 1090 strain (AE004969.1), and shared 95–96% homology with *Neisseria meningitidis* homologous gene omp4. Regardless, the homologies of Omp3 (Rmp) protein amino acid sequences in all the six *N. gonorrhoeae* strains were 100%, indicating that the protein was highly conserved. Thus, the Rmp deletion mutant strain might have wide application in the study of novel gonorrhea live attenuated vaccines.

Gonococci escape host immunity is associated with the immune blocking of Rmp as well as the immunosuppression caused by Opa proteins [40]–[41]. Binding of Opa proteins to carcinoembryonic antigen-related cellular adhesion molecule 1 (CEACAM1 or CD66a) kills human peripheral B cells, inhibits antibody production, and arrests the activation and proliferation of CD4+ T lymphocytes [40]–[41]. In the present study, both the mutant and wild-type strains were capable of inducing high-titer antibodies in mice since mCEACAM1 cannot bind *N. gonorrhoeae* [42]. The Rmp deletion mutant strain constructed herein is feasible for developing novel live attenuated vaccines for gonorrhea by Opa genes deletion or screening of phenotypic variant strains that do not express Opa proteins [43].

## Supporting Information

Table S1 **Bacteria survival rates (%, n = 4), means, standard deviations and the difference of variances of the 4 groups at each sera 1/titer (results of One-way ANOVA by SPSS10.0).**
(DOC)

Table S2 **The P-values at each sera 1/titer for 3 of the pairwise comparisons analyzed by Bonferroni correction.**
(DOC)
