# Supplementary material for: Antibodies with Higher Bactericidal Activity Induced by a Neisseria gonorrhoeae Rmp Deletion Mutant Strain
Source: PLoS One. 2014 Mar 4;9(3):e90525. doi: 10.1371/journal.pone.0090525 (PMC3942440; doi:10.1371/journal.pone.0090525)
Supplement: Table S1 — Bacteria survival rates (%, n = 4), means, standard deviations and the difference of variances of the 4 groups at each sera 1/titer (results of One-way ANOVA by SPSS10.0). (DOC) [file pone.0090525.s001.doc]

| **1/titer** | | **anti-PorB+anti-rRmp** | **anti-WT** | **anti-porB** | **anti-MT** | **Homogeneity of Variances (Sig.)** |
| --- | --- | --- | --- | --- | --- | --- |
| 0 | 1 | 101.025 | 103.36 | 102.086 | 98.38 | **0.053** |
|  | 2 | 98.667 | 104.824 | 98.655 | 97.596 |  |
|  | 3 | 99.45 | 90.98 | 99.322 | 102.599 |  |
|  | 4 | 100.858 | 100.836 | 99.937 | 101.425 |  |
|  | Mean | 100 | 100 | 100 | 100 |  |
|  | Std. Deviation | 1.135 | 7.746 | 1.486 | 2.394 |  |
| 0.000064 | 1 | 96.244 | 89.958 | 94.356 | 85.005 | **0.272** |
|  | 2 | 94.76 | 94.622 | 93.703 | 76.575 |  |
|  | 3 | 91.566 | 84.052 | 90.208 | 88.86 |  |
|  | 4 | 92.902 | 92.326 | 94.665 | 81.665 |  |
|  | Mean | 93.868 | 90.24 | 93.233 | 83.026 |  |
|  | Std. Deviation | 2.055 | 4.543 | 2.056 | 5.21 |  |
| 0.00032 | 1 | 96.452 | 89.66 | 84.005 | 81.55 | **0.173** |
|  | 2 | 93.57 | 84.908 | 83.2 | 71.962 |  |
|  | 3 | 87.608 | 89.445 | 80.685 | 80.221 |  |
|  | 4 | 89.485 | 86.202 | 82.88 | 78.458 |  |
|  | Mean | 91.779 | 87.554 | 82.693 | 78.048 |  |
|  | Std. Deviation | 3.988 | 2.369 | 1.42 | 4.25 |  |
| 0.0016 | 1 | 90.01 | 83.865 | 77.667 | 57.776 | **0.357** |
|  | 2 | 85.465 | 80.56 | 70.25 | 65.85 |  |
|  | 3 | 94.552 | 85.255 | 81.334 | 48.643 |  |
|  | 4 | 81.883 | 81.408 | 72.505 | 54.886 |  |
|  | Mean | 87.978 | 82.772 | 75.439 | 56.789 |  |
|  | Std. Deviation | 5.502 | 2.169 | 5.009 | 7.143 |  |
| 0.008 | 1 | 79.776 | 64.77 | 70.084 | 40.055 | **0.792** |
|  | 2 | 73.225 | 60.25 | 67.65 | 47.576 |  |
|  | 3 | 81.565 | 71.785 | 78.812 | 35.42 |  |
|  | 4 | 78.48 | 66.005 | 69.112 | 44.925 |  |
|  | Mean | 78.262 | 65.703 | 71.415 | 41.994 |  |
|  | Std. Deviation | 3.588 | 4.75 | 5.032 | 5.377 |  |
| 0.04 | 1 | 75.45 | 56.88 | 51.656 | 43.655 | **0.673** |
|  | 2 | 80.802 | 61.885 | 57.55 | 44.38 |  |
|  | 3 | 86.665 | 59.945 | 60.875 | 38.866 |  |
|  | 4 | 77.304 | 65.422 | 57.465 | 43.334 |  |
|  | Mean | 80.055 | 61.033 | 56.887 | 42.559 |  |
|  | Std. Deviation | 4.934 | 3.579 | 3.831 | 2.5 |  |
| 0.2 | 1 | 64.68 | 52.544 | 40.445 | 10.667 | **0.284** |
|  | 2 | 60.552 | 60.66 | 44.978 | 14.365 |  |
|  | 3 | 69.883 | 41.404 | 48.667 | 22.75 |  |
|  | 4 | 63.335 | 58.886 | 46.85 | 18.506 |  |
|  | Mean | 64.613 | 53.374 | 45.235 | 16.572 |  |
|  | Std. Deviation | 3.912 | 8.707 | 3.531 | 5.217 |  |
| 1 | 1 | 53.54 | 41.655 | 38.65 | 11.182 | **0.615** |
|  | 2 | 60.56 | 48.992 | 23.578 | 4.334 |  |
|  | 3 | 63.458 | 35.055 | 44.54 | 15.65 |  |
|  | 4 | 58.395 | 43.65 | 36.85 | 11.856 |  |
|  | Mean | 58.988 | 42.338 | 35.905 | 10.756 |  |
|  | Std. Deviation | 4.183 | 5.759 | 8.85 | 4.711 |  |
