# Supplementary material for: Antibodies with Higher Bactericidal Activity Induced by a Neisseria gonorrhoeae Rmp Deletion Mutant Strain
Source: PLoS One. 2014 Mar 4;9(3):e90525. doi: 10.1371/journal.pone.0090525 (PMC3942440; doi:10.1371/journal.pone.0090525)
Supplement: Table S2 — The P-values at each sera 1/titer for 3 of the pairwise comparisons analyzed by Bonferroni correction. (DOC) [file pone.0090525.s002.doc]

| **1/titer** | **anti-MT^a^ vs. anti-WT** | **anti-MT^a^ vs. anti-PorB** | **anti-porB^b^ vs. anti-PorB+anti-Rmp** |
| --- | --- | --- | --- |
| 0 | 1.000 | 1.000 | 1.000 |
| 0.000064 | 0.112 | 0.0139 | 1.000 |
| 0.00032 | 0.00781 | 0.386 | 0.0109 |
| 0.0016 | 0.0000894 | 0.00184 | 0.0337 |
| 0.008 | **0.0000768** | **0.0000085** | 0.38 |
| 0.04 | **0.000105** | **0.0011** | 0.0000107 |
| 0.2 | **0.00000588** | **0.000076** | **0.00264** |
| 1 | **0.0000596** | **0.00052** | **0.00111** |
